# Adult Onset Acute Disseminated Encephalomyelitis: A Case Report

**DOI:** 10.7759/cureus.72487

**Published:** 2024-10-27

**Authors:** João R Corrêa, Ana P Silva, Joana Coelho, Renato Gonçalves, Dália Estevão

**Affiliations:** 1 Internal Medicine, Centro Hospitalar Universitário Cova da Beira, Covilhã, PRT

**Keywords:** acute ataxia, acute disseminated encephalomyelitis (adem), adult-onset, • encephalopathy, vitamin-d deficiency

## Abstract

Acute disseminated encephalomyelitis (ADEM) is a rare autoimmune demyelinating disorder of the central nervous system that can mimic other neurological diseases, such as multiple sclerosis. ADEM is thought to manifest in the presence of environmental triggers, namely viral or bacterial infections, with multiple simultaneous neurological deficits, frequently accompanied by encephalopathy. Here, we report the case of a 49-year-old female patient who presented in the emergency department with encephalopathy, right-side muscle weakness, dizziness, vertigo, ataxia, and postural imbalance, preceded by symptoms suggesting recent pharyngitis/sialadenitis three weeks prior. Cerebrospinal fluid analysis revealed lymphocytic pleocytosis, elevated protein levels, normal glucose levels, no oligoclonal bands, and culture and viral studies were negative. After a normal cerebral computed tomography, brain and cervical spine magnetic resonance imaging (MRI) revealed multiple, T2-weighted hyperintense supratentorial and infratentorial white matter lesions, including the right cerebellar peduncle and posterior limb of the left internal capsule. The diagnosis of ADEM was made, and the patient was treated with high-dose intravenous glucocorticoids followed by oral tapering with clinical improvement. During follow-up, the control MRI was compatible with the diagnosis. This case illustrates the diagnostic approach of a patient presenting with subacute neurological deficits and the importance of contemplating possible differential diagnoses and swiftly initiating treatment.

## Introduction

Acute disseminated encephalomyelitis (ADEM) is a rare autoimmune demyelinating disease of the central nervous system, affecting the brain and spinal cord [1].

Although the pathogenesis is not completely understood, it seems to affect genetically susceptible individuals after an environmental trigger. Preceding viral or bacterial infection, present in up to 50% of patients, is the most frequent and well-described trigger associated with ADEM [2]. A relationship between ADEM and immunization has also been reported but remains controversial [3].

Because of this association with viral infections and immunization, ADEM is more frequent in children, with an estimated annual incidence of five cases per one million inhabitants [1,4,5].

ADEM typically manifests as an acute and often rapidly deteriorating condition, causing multifocal neurologic symptoms and encephalopathy [2,6]. Motor and sensory deficits, as well as brainstem involvement causing oculomotor deficits and dysarthria, are common [2]. Additional signs and symptoms include headache, ataxia, aphasia, nystagmus, extrapyramidal symptoms, urinary retention, seizures, and increased intracranial pressure [6,7].

## Case presentation

A 49-year-old female patient with a past medical history of hypertension, dyslipidemia, current smoking, depression, and hypothyroidism presented to the emergency department (ED) with dizziness, vertigo, muscle weakness, postural imbalance, and an abnormal gait. At observation, the symptoms were present for the last two weeks, without signs of improvement.

Three weeks before symptoms started, the patient was observed by her primary care physician for fever, dry cough, odynophagia, and infra-mandibular swelling. According to the records, there was the presence of pus on oropharynx observation, and she improved after treatment with oral antibiotics. She reported no other prior symptoms or travels. On the neurological examination, the patient showed drowsiness, confusion, bilaterally impaired smooth pursuit eye movement, right upper limb (grade 3) and right lower limb (grade 4) muscle weakness, ataxia, and postural disequilibrium. During her hospital stay, she also reported episodes of urinary incontinence. No other neurological signs were found.

Blood analysis (Table 1) showed mild leucocytosis (10.6 x 10^3^/uL) and neutrophilia (8.3 x 10^3^/uL), a slight increase in C-reactive protein (1.08 mg/dL), and an elevation of the erythrocyte sedimentation rate (41 mm/H).

**Table 1:** Laboratory values of blood analysis at admission.

Laboratory parameter	Value (Reference range)	Units
White blood cell count	10.6 (4.0 – 10.0) x 10^3^	Cells/microliter
Neutrophils	8.3 (1.5 – 8.0) x 10^3^	Cells/microliter
Lymphocytes	1.6 (0.8 – 4.0) x 10^3^	Cells/microliter
Hemoglobin	13.5 (13.6 – 18.0)	g/dL
Platelets	200 (150 – 450) x 10^3^	Cells/microliter
Creatinine	0.67 (0.70 – 1.20)	mg/dL
Blood urea nitrogen	13 (8 – 22)	mg/dL
C-reactive protein	1.08 (0.00 – 0.50)	mg/dL
Erythrocyte sedimentation rate	41 (0 – 15)	mm/H

Cerebrospinal fluid (CSF) analysis (Table 2) revealed lymphocytic pleocytosis (white blood cell count of 17.4 cells/mm^3^, with 99% being lymphocytes), elevated CSF protein levels (120.4 mg/dL), normal glucose levels, and no oligoclonal bands. CSF culture and viral studies were negative.

**Table 2:** Laboratory values of cerebrospinal fluid analysis.

Laboratory parameter	Value (Reference range)	Units
Glucose	56 (40 – 76)	mg/dL
Proteins	120.4 (10.0 – 45.0)	mg/dL
IgG	5.76 (0.48 – 5.86)	mg/dL
White blood cell count	17.4 (< 5)	Cells/mm^3^

**Figure 1:**
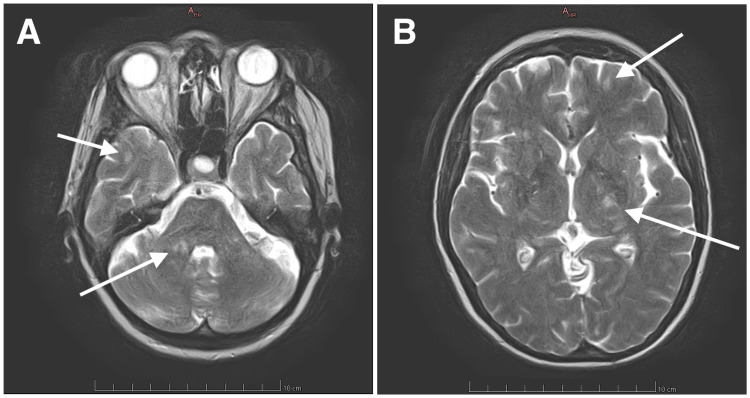
Initial cerebral MRI exhibiting multiple T2-weighted hyperintense white matter lesions (arrows) on the middle right cerebellar peduncle and right temporal lobe (Panel A) and posterior limb of the left internal capsule and left frontal lobe (Panel B).

Initial cerebral computed tomography (CT) scans and CT angiography exhibited no abnormalities. Cerebral magnetic resonance imaging (MRI) made apparent multiple supratentorial and infratentorial white matter lesions, displaying T2-weighted hyperintensity, namely in the middle right cerebellar peduncle, both cerebral peduncles, posterior limb of the internal capsule, corpus callosum, corona radiata, and centrum semiovale, with the lesions in the corpus callosum and posterior limb of the left internal capsule showing moderate diffusion restriction (Figure 1). Cervical spine MRI showed no abnormalities.

Anti-aquaporin-4 (anti-AQP4) and anti-myelin oligodendrocyte glycoprotein (anti-MOG) antibodies were negative, and no evidence, clinical or serologic, of systemic autoimmune disease was found. Other than vitamin D deficiency (17.6 ng/mL), no other metabolic or endocrine abnormalities were present (normal thyroid levels with medication). Infections with COVID-19, human immunodeficiency virus, B or C hepatitis, Listeria monocytogenes, or Borrelia burgdorferi were also excluded.

Considering the clinical features (presence of encephalopathy, multiple concurrent neurologic deficits, recent medical history suggestive of preceding infection, absence of oligoclonal bands (OCBs) in the CSF analysis) and MRI findings (bilateral and asymmetric lesions with poorly defined margins and of similar age, and absence of hypointense T1-weighted lesions), a presumptive diagnosis of acute disseminated encephalomyelitis was made.

The patient was started on intravenous corticoid therapy with 1000 mg daily of methylprednisolone for five days, associated with encephalopathy resolution and improvement in postural balance, ataxia, and gait capability. She was discharged with tapering oral corticosteroid therapy and physical rehabilitation.

**Figure 2:**
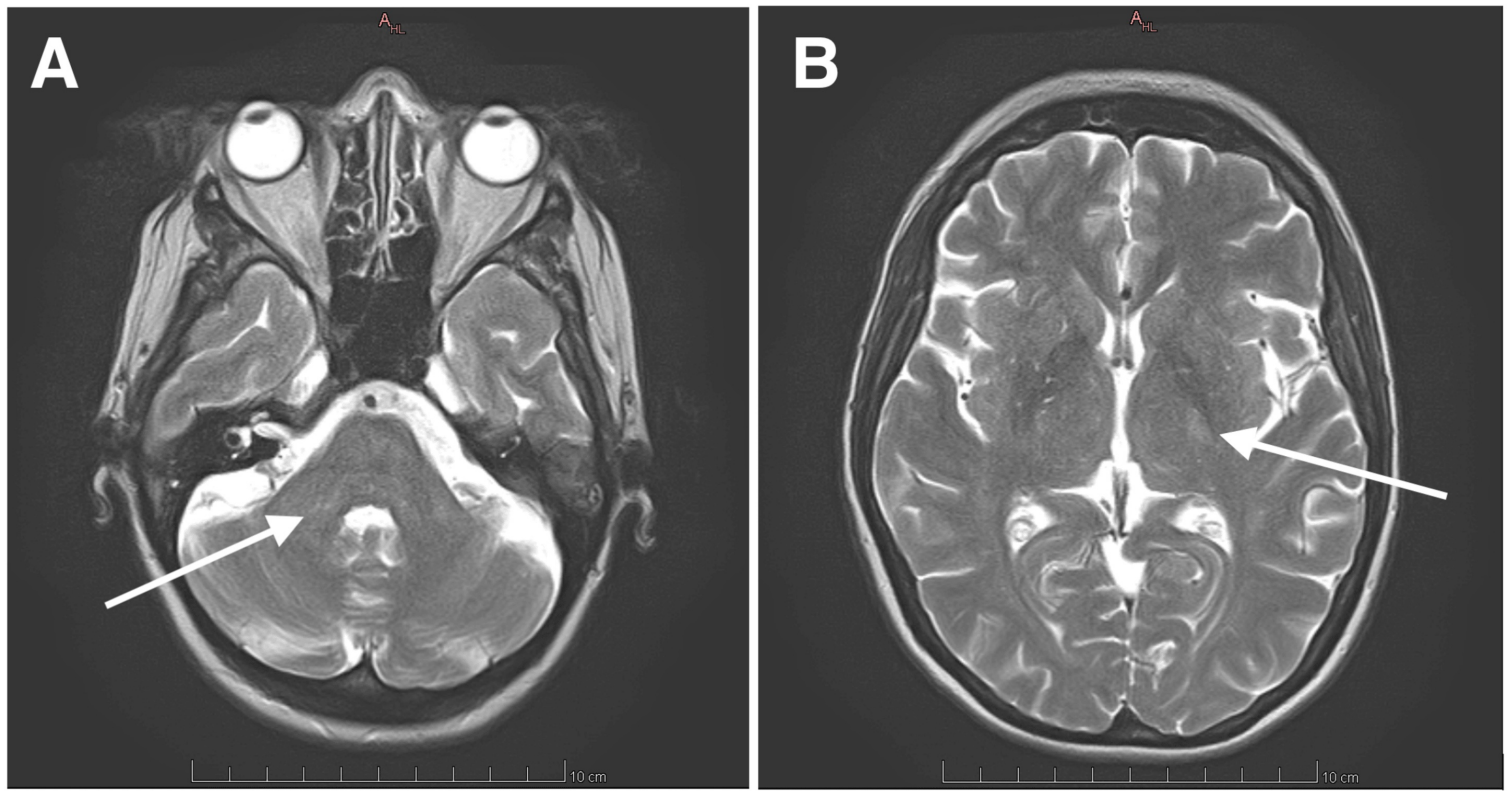
Six-month cerebral MRI with minimal T2-weighted hyperintense lesion on the middle right cerebellar peduncle (arrow, Panel A) and residual lesion on the posterior limb of the left internal capsule (arrow, Panel B) displaying less T2-weighted hyperintensity compared with Figure 1.

A cerebral MRI performed six months after discharge showed a reduction in the number, size, and T2 hyperintensity of the previously documented lesions, without new or T1 hypointense lesions, highly suggestive of ADEM (Figure 2). At the one-year mark after discharge, the only neurological deficit remaining was intermittent urinary incontinence.

## Discussion

The most challenging aspect of the diagnostic process in ADEM is the differential diagnosis with a first attack of multiple sclerosis (MS), myelin oligodendrocyte glycoprotein antibody-associated disorder (MOGAD), or neuromyelitis optica spectrum disorder (NMOSD). Central nervous system (CNS) infections, vasculitis, neurologic sarcoidosis, and Behçet disease are other entities to consider.

Therefore, the diagnosis of ADEM requires high clinical suspicion based on the presence of multifocal neurological signs and trigger identification; exclusion of other causes such as infectious diseases or vasculitis; and identification of typical lesions on MRI, bilateral, asymmetric, poorly marginated T2-weighted hyperintense lesions, with the same evolution time, and absent or inconspicuous T1-weighted hypointense lesions [8,9].

In ADEM, abnormalities in CSF analysis are common (present in 50-80% of patients) but nonspecific (lymphocytic pleocytosis and mildly elevated CSF protein level) [2,10]. Our patient had both of these findings, but their CSF protein level was greater (120.4 mg/dL) than the value usually described in ADEM (<70 mg/dL) [10,11]. CSF analysis is particularly important in ruling out other diagnoses, such as central nervous system (CNS) infections.

Oligoclonal bands (OCBs) can also be found in the CSF of up to 25% of patients with ADEM. However, when present, they are usually identical to serum OCBs, which suggests a systemic immune system activation, contrary to the CSF-specific OCB pattern found in multiple sclerosis (MS) [2]. In our patient, OCBs were absent in CSF analysis; therefore, we did not test for the presence of serum OCBs.

Considering the absence of anti-aquaporin-4 (anti-AQP4) or anti-myelin oligodendrocyte glycoprotein (anti-MOG) antibodies in our patient, as well as the absence of clinical or serologic evidence for other systemic autoimmune disease, the leading differential diagnosis in our patient is MS.

MS is a progressive demyelinating disease involving the central nervous system that generally follows a relapsing-remitting course, with each attack usually manifesting as a focal neurologic deficit [12]. Contrary to ADEM, encephalopathy and/or impaired consciousness or preceding infection are uncommon features [7,8]. The presence of encephalopathy and multifocal deficits, absence of OCBs in the CSF analysis, and recent medical history compatible with an infectious trigger are more suggestive of ADEM [7,8].

The MRI findings of multiple bilateral and asymmetric lesions, with poorly defined margins and the same evolution time, and the absence of hypointense T1-weighted lesions (black holes) in a 49-year-old patient strengthen the diagnosis of ADEM [9,13]. MS lesions tend to have better-defined margins and different ages, frequently with the presence of black holes [9]. Furthermore, the improvement evidenced during the control MRI, six months after discharge, and the absence of new lesions or black holes is also highly suggestive of this hypothesis [12,13].

High-dose intravenous glucocorticoid therapy (1000 mg methylprednisolone for three to five days), followed by an oral glucocorticoid taper over four to six weeks, is the first-line therapy recommended for adults with ADEM and was associated with clinical improvement in the majority of patients, as seen in our case [11,14]. In non-responsive or poorly responsive patients, other diagnoses should be considered, and alternative options include intravenous immune globulin, plasma exchange, and cyclophosphamide [15,16].

Appropriate and expeditious treatment is crucial for clinical improvement. Nonetheless, adult populations are more prone to severe clinical courses and less favorable outcomes, and complete recovery is reported in less than 50% of the cases [2,8,11]. At a one-year follow-up, our patient still displayed intermittent episodes of urinary incontinence. Complete spinal evaluation in the next control MRI, as well as further urological evaluation, will help to better understand its etiology.

During childhood, our patient received the measles-mumps-rubella vaccine, but taking into account the preceding symptoms of fever, odynophagia, and infra-mandibular swelling, serologic testing for mumps could have been done to discard this hypothesis.

Finally, we have found our patient to be vitamin D deficient. The immunoregulatory effects of vitamin D have been well established in the literature, with vitamin D deficiency being associated with an increasing risk of immune-mediated and inflammatory diseases, such as MS [17,18]. A retrospective study by Tomoum HY et al. has also found a link between vitamin D deficiency and the risk of relapse in children with acute demyelinating disorders, including ADEM, but using a small sample (20 children, seven of whom had ADEM) [19].

In the authors’ view, more studies are needed to confirm this association, including studies in adult populations, but we recommend testing and treating for vitamin D deficiency in patients with ADEM, especially in areas where vitamin D deficiency is endemic [17,19].

## Conclusions

Acute disseminated encephalomyelitis is a rare immune-mediated inflammatory disease that can mimic other neurological and systemic diseases, particularly multiple sclerosis. The hallmark clues that can help differentiate ADEM from other conditions include recent infection, presence of encephalopathy, multiple neurological signs or symptoms at presentation, and brain or spinal MRI findings (bilateral, asymmetric, poorly marginated T2-weighted hyperintense lesions with the same evolution time; absent or inconspicuous T1-weighted hypointense lesions; imaging improvement or resolution in control MRI). It is of utmost importance that clinicians are acquainted with this disease in order to establish the correct diagnosis, treatment, and follow-up plan, especially in adults. Treatment with high doses of intravenous glucocorticoids followed by oral tapering is associated with clinical improvement, but complete recovery occurs in less than 50% of adult populations.
